# Serotype distribution and incidence of invasive early onset and late onset group B streptococcal disease amongst infants in Singapore

**DOI:** 10.1186/s12879-021-06891-1

**Published:** 2021-12-07

**Authors:** Kai-Qian Kam, Koh Cheng Thoon, Wen Sim Nancy Tee, Michelle Lay Teng Ang, Natalie Woon Hui Tan, Kee Thai Yeo, Jiahui Li, Chia Yin Chong

**Affiliations:** 1 Infectious Disease Service, Department of Pediatrics, KK Women’s and Children’s Hospital, 100 Bukit Timah, Singapore City, 229899 Singapore; 2 Yong Loo Lin School of Medicine, National University of Singapore, Singapore City, Singapore; 3 Duke-National University of Singapore Medical School, Singapore City, Singapore; 4 Lee Kong Chian School of Medicine, Nanyang Technological University, Singapore City, Singapore; 5 Department of Laboratory Medicine, National University Health System, Singapore City, Singapore; 6 National Public Health Laboratory, National Centre of Infectious Diseases, Singapore City, Singapore; 7 Department of Neonatology, KK Women’s and Children’s Hospital, Singapore City, Singapore

**Keywords:** GBS, Group B streptococcus, Serotype, Incidence, Risk factors, EOD, LOD, Singapore

## Abstract

**Background:**

The current group B streptococcal (GBS) preventive measures had reduced invasive GBS early onset disease (EOD) incidences worldwide, but the late onset disease (LOD) incidences had remained unchanged. Administration of a safe and effective GBS vaccine in addition to the current strategies were thought to be the next steps in reducing the incidences of invasive GBS infection especially LOD. In this study, we aimed to examine the causative GBS serotypes in invasive GBS disease, determine the incidences of EOD and LOD, and compare the risk factors between EOD and LOD.

**Methods:**

A retrospective study of infants ≤ 90-day-old over an 8-year period (2010–2017). The incidences of EOD and LOD were obtained by using patients with EOD and LOD who were born in our institution as the numerator and the live births in our institution per year of the study period as the denominator. Available GBS isolates were serotyped by the National Public Health Laboratory using capsular serotyping methods. The risk factors of EOD and LOD were compared.

**Results:**

A total of 71 infants were identified; 16 (22.5%) and 55 (77.5%) of them had EOD and LOD, respectively. Serotype III (n = 42, 71.2%) was the most common serotype amongst the 59 isolates available for serotyping. Serotypes Ia, Ib, II, III, and V accounted for 98.3% (n = 58) of the invasive GBS diseases. The overall incidence was 0.42 per 1000 live births. The mean incidences of EOD and LOD were 0.13 per 1000 live births and 0.29 per 1000 live births, respectively. On multivariate analysis, risk factors for LOD as compared to EOD were: Chinese ethnicity (OR 27.1, 95% CI 3.0–243.1, p = 0.003) and negative/unknown maternal GBS status (OR 20.0, 95% CI 2.0–250.0, p = 0.012). Prematurity and intrapartum risk factors (peripartum maternal pyrexia, prolonged rupture of membrane) of EOD were not associated with LOD.

**Conclusions:**

The LOD incidence had remained higher than EOD incidence in our cohort. A GBS vaccine that covers the major causative serotypes found in our cohort can potentially reduce the overall GBS disease burden in the country.

## Background

Lancefield group B Streptococcus (GBS) or *Streptococcus agalactiae* invasive disease continues to cause significant mortality and morbidity in neonates and young infants. In 1996, the Centres for Disease Control and Prevention (CDC) published the consensus guideline on the prevention of perinatal GBS infection and this was subsequently updated in 2019. Based on this guideline, CDC advocated for universal screening of all pregnant women at 35 to 37 weeks of gestation for maternal vaginal and rectal GBS colonization [1, 2]. It was also recommended that intrapartum antibiotic prophylaxis should be given to the pregnant women who were screened positive for GBS colonization or had other risk factors [1, 2]. With the implementation of these guidelines, the incidence of GBS early onset disease (EOD) in the United States of America (USA) had decreased from 1.8 cases per 1000 live births in 1990 to 0.23 cases per 1000 live births in 2015 [3]. Comparatively, the incidence of GBS late onset disease (LOD) had been stable at an average of 0.31 cases per 1000 live births from 2006 to 2015. While the risk factors for GBS EOD are well studied and proven, the risk factors for LOD are less clear although preterm birth had been described to be strongly associated with LOD [2–6].

In 2010, our institution had adopted specific steps to reduce the incidence of invasive GBS disease in our hospital based on recommendations from Royal College of Obstetricians and Gynaecologists (RCOG), CDC and National Institute for Health and Care Excellence (NICE) guidelines [1, 7, 8]. Vaginal GBS screening of pregnant women was conducted at 35 to 37 weeks if there was no planned elective Caesarean section. Indications for intrapartum GBS prophylaxis in the form of intravenous penicillin included any of the following: (i) previous infant with invasive GBS disease (ii) GBS bacteriuria during the current pregnancy (iii) positive GBS screening test during current pregnancy (iv) unknown GBS status and delivery at < 37 weeks gestation or rupture of membranes > 18 h or intrapartum temperature ≥ 38.0 °C. For patients where Caesarean section was performed before the labour onset or with intact amniotic membranes, intrapartum GBS prophylaxis was not indicated.

In a previous study done in our institution from 1996 to 1997 where the routine screening for maternal GBS status and administration of intrapartum antibiotics to high-risk pregnant women were not the standard of practice, the GBS EOD and LOD incidences were reported to be 0.265 per 1000 live births and 0.07 per 1000 live births, respectively [9]. There were no further studies that looked at the impact of the implementation of the guidelines on the EOD and LOD incidences after 2010. Data regarding the incidences of GBS EOD and LOD in Singapore and other Asia countries were also limited [10].

With a recent hexavalent capsular polysaccharide conjugate vaccine GBS6 (serotypes Ia, Ib, II, III, IV and V) that was undergoing trials [11], it has been proposed that administration of a safe and effective GBS vaccine in addition to the current GBS prevention strategies may further decrease the incidence of invasive GBS infection especially LOD [12]. We sought to understand the potential serotype coverage of this GBS conjugate vaccine in our population. In this study, we aimed to examine the causative GBS serotypes in invasive GBS disease, determine the incidences of EOD and LOD, and compare the risk factors between EOD and LOD.

## Methods

### Setting

KK Women’s and Children’s Hospital (KKH) is the largest tertiary pediatric hospital in Singapore with more than 11,000 live births per year with approximately 500 pediatric/neonatal inpatient beds. Our institution also houses 48.5% of Singapore’s neonatal and pediatric admissions with approximately 32,000 pediatric admissions per year (data from Ministry of Health, Singapore). Approval for waiver of consent was obtained from the centralized institution review board of SingHealth Research (Reference number 2017/2031).

### Study design

In this retrospective cohort study, we included infants from birth to day 90 of life who were diagnosed with invasive GBS disease from January 2010 to October 2017 over an 8-year period. A list of patients with GBS isolated from the blood, cerebrospinal fluid (CSF) or other sterile body site cultures was obtained from the KKH microbiology department. Demographic characteristics, the type of invasive GBS disease, perinatal and antenatal risk factors were evaluated from the case notes and electronic medical records.

### Laboratory methods

Before January 2014, bacterial strains from blood and sterile site cultures were isolated on blood agar plates. Bacterial identification was based on Lancefield grouping, microscopic and colony morphology. From January 2014 onwards, isolates were identified using MALDI-TOF mass spectrometry (bioMerieux Vitek MS). Quality control for the Vitek MS was performed by using an ATCC control strain (*Escherichia coli* ATCC 8739) and performed daily as recommended by the manufacturer. Capsular typing was conducted by the National Public Health Laboratory (NPHL) of the Ministry of Health, Singapore. Capsular serotyping involved the use of several sets of multiplex polymerase chain reaction (PCR), each containing primer pairs specific for the various capsular serotypes. Serotype assignment was based on the amplicon size obtained. Each strain was initially subjected to 2 multiplex PCR [13]. PCR primer mix 1 contained primer pairs specific for capsular polysaccharide types Ia, Ib, II, III, and IV and PCR mix 2 contained primer pairs specific for types V, VI, VII or IX, and VIII. The amplicons obtained were visualised by gel electrophoresis. Strains assigned to serotype VII were further subjected to a third multiplex PCR to distinguish between serotype VII and IX [14]. All the GBS isolates except for those from year 2014 were available for capsular typing. Sequence types were determined by using multi-locus sequence typing [15, 16]. Conventional PCR for the loci of interest was performed. This was followed by Sanger sequencing of the amplicons obtained. Sequences were later compared against the Streptococcus agalactiae multi-locus sequence typing (MLST) database to obtain the sequence types.

### Study definitions

GBS EOD and GBS LOD were defined as isolation of GBS from a sterile site from birth to day 6 of life and from day 7 of life to day 90 of life, respectively [2]. GBS bacteremia was defined as growth of GBS from blood culture of the infant. GBS meningitis was defined as growth of GBS from the CSF culture, or a positive CSF latex agglutination test, but negative CSF culture with a positive blood culture for GBS. The incidences of EOD and LOD were obtained by using patients with EOD and LOD who were born in KKH as the numerator and the live births at KKH per year of the study period as the denominator.

### Statistical analysis

Statistical analysis was performed using SPSS statistical program version 19 (SPSS, Inc, Chicago, Illinois). Risk factors were compared between EOD and LOD using Chi-square test or Fisher’s exact test (when numbers were < 5) for categorical variables. Variables with p values of < 0.2 on univariate analysis were selected for multivariate analysis via binary logistic regression to identify risk factors that predicted LOD. All statistical tests were two-tailed and a p value < 0.05 was statistically significant.

## Results

### Patient’s characteristics

Seventy-one infants with invasive GBS disease were identified; 16 (22.5%) of them had EOD, 55 (77.5%) of them had LOD. Thirty-nine infants (54.9%) were born in KKH while the remaining were born in other hospitals. The median age where EOD and LOD had occurred was 1 day-old (interquartile range [IQR] = 1–3) and 34 day-old (IQR = 17–64), respectively. Bacteremia was the predominant invasive disease in 76.1% (n = 54) of the infants (Table 1). There was a larger percentage of infants with LOD who presented with meningitis (with or without concomitant bacteremia) (n = 14, 25.5%) as compared to EOD (n = 1, 6.3%). None of the infants in our cohort had received outpatient antibiotics for their febrile illness prior to the presentation to the hospital. The infants’ mothers did not have any chronic medical conditions and they were not on immunosuppressive therapies prior to delivery. All the mothers tested negative for human immunodeficiency virus (HIV) during the antenatal period. A total of 37 infants’ mothers (52.1%) were screened for GBS during pregnancy; 10 mothers were determined to have GBS infection or colonization, and 27 mothers were screened negative for GBS.

**Table 1:** Clinical presentation and risk factors of infants with invasive GBS Infection in KKH, Singapore (2010–2018)

Patient characteristics	LOD^a^ (n = 55)	EOD^a^ (n = 16)	Univariate analysis			Multivariate analysis^b^		
			Odds ratio	95% CI	p value	Odds ratio	95% CI	p value
Day of life when GBS infection occurred, median (IQR)	34 (17–64)	1 (1–3)	NA			NA		
Type of disease			NA			NA		
Bacteremia only	40 (72.7)	14 (87.5)						
Meningitis only	2 (3.6)	0						
Bacteremia and meningitis	12 (21.8)	1 (6.3)						
Bacteremia, endophthalmitis and arthritis	0	1 (6.3)						
Arthritis	1 (1.8)	0						
Mode of delivery^c^			0.2	0.05–1.1	0.058	–		0.138
Normal or assisted vaginal delivery	33 (60.0)	14 (87.5)						
Caesarean section	20 (36.4)	2 (12.5)						
Race			15.6	3.2–76.6	< 0.001	27.1	3.0–243.1	0.003
Chinese	38 (69.1)	2 (12.5)						
Non-Chinese	17 (30.9)	14 (87.5)						
Gender			1.0	0.3–3.1	0.961	NA		
Male	34 (61.8)	10 (62.5)						
Female	21 (38.2)	6 (37.5)						
Gestational age			0.4	0.1–1.2	0.081	–		0.480
Preterm^d^	12 (21.8)	7 (43.8)						
Term	43 (78.2)	9 (56.3)						
Birth weight			2.7	0.8–9.2	0.104	–		0.555
Normal birth weight	45 (81.8)	10 (62.5)						
Low birth weight	10 (18.2)	6 (37.5)						
Maternal GBS status			10.0	2.0–33.3	0.007	20.0	0.004–0.5	0.012
Negative or unknown	51 (92.7)	10 (62.5)						
Infection or colonization	4 (7.3)	6 (37.5)						
Adequate intrapartum antibiotics			3.5	1.0–12.4	0.042	–		0.637
Yes or not required	47 (85.5)	10 (62.5)						
No	8 (14.5)	6 (37.5)						
Prolonged rupture of membrane^e^			NA^f^	NA^f^	0.267	NA		
Yes	4 (7.3)	0 (0)						
No	51 (92.7)	16 (100)						
Peripartum maternal pyrexia			0.2	0.03–1.1	0.072	–		0.759
Yes	2 (3.6)	3 (18.8)						
No	53 (96.4)	13 (81.3)						
Serotypes^g^			5.3	1.5–19.3	0.007	–		0.099
III	36 (65.5)	6 (37.5)						
Others	9 (16.4)	8 (50.0)						

*KKH* KK Women’s and Children’s Hospital, *LOD* late onset disease, *EOD* early onset disease, *IQR* interquartile range, *GBS* group B  streptococcus, *NA* not applicable

^a^Unless otherwise indicated, data are expressed as number (percentage) of patients

^b^Multivariate analysis included the following variables: race, mode of delivery, gestational age, birth weight, maternal GBS status, adequate antepartum antibiotics, maternal pyrexia, serotypes. Gender was not included as logistic regression was done for variables on univariate analysis where p < 0.2

^c^2 missing data for LOD

^d^Preterm is defined as gestational age of less than 37 weeks

^e^Prolonged rupture of membrane is defined as rupture of membrane for more than 18 h before delivery

^f ^Odds ratio is invalid as there were no EOD cases with prolonged rupture of membrane

^g^12 missing data; 10 and 2 isolates were not serotyped for LOD and EOD, respectively

### Clinical course and outcome

All infants survived the GBS invasive disease episode, and no infants were readmitted for recurrence of GBS disease during the study period. In our cohort, GBS bacteremia was treated with an average of 10 to 14 days of intravenous penicillin/ampicillin or ceftriaxone/cefotaxime while GBS meningitis required an average of 21 days of penicillin/ampicillin or ceftriaxone with or without 5 days of gentamicin for synergistic effect. Two neonates with LOD had meningitis with subdural collection and required 6 to 8 weeks of antibiotic treatment. They did not have any surgical intervention. Another neonate with EOD had GBS bacteremia with right hip septic arthritis, left endophthalmitis and urinary tract infection on day 6 of life. He underwent right hip arthrotomy and washout, and left lensectomy. The fluid culture from the right hip and left vitreous fluid were positive for GBS.

Three neonates with EOD and 1 neonate with LOD were admitted to the Intensive Care Unit for septic shock requiring inotropic support and had multi-organ dysfunction. Amongst these four infants, one neonate underwent 5 days of extracorporeal membrane oxygenation (ECMO) for intractable septic shock.

### Serotype distribution

**Fig. 1:**
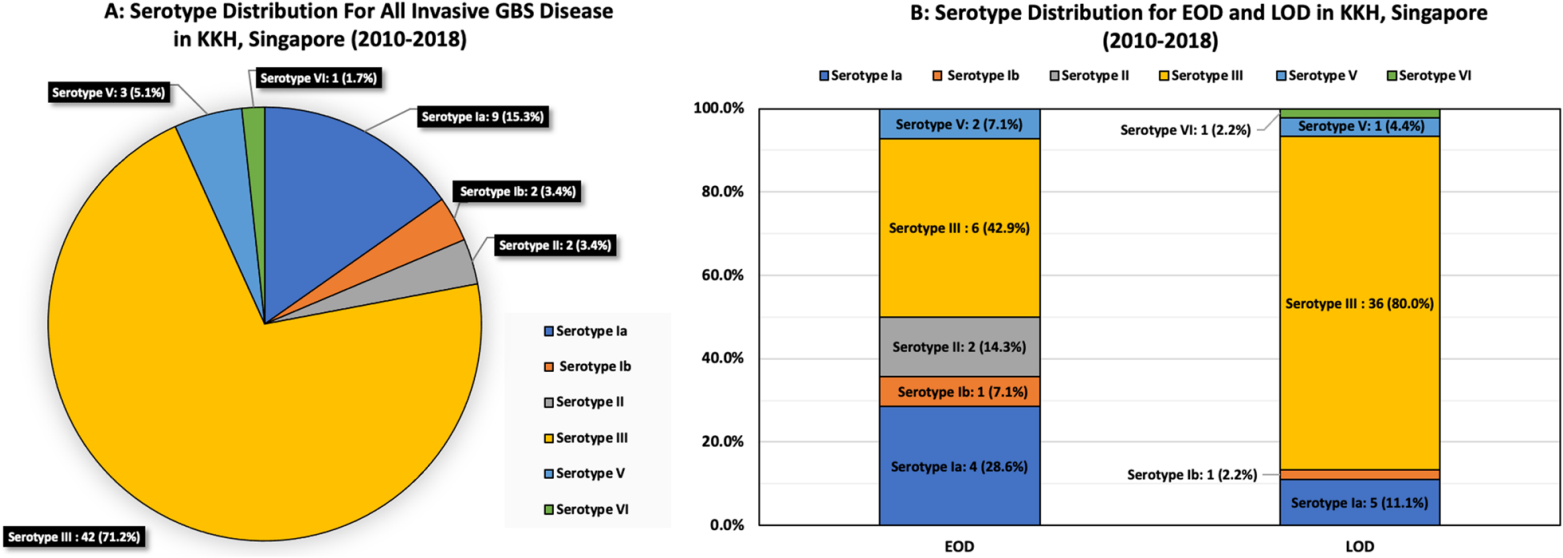
Overall serotype distribution for GBS disease detected in KKH, Singapore, from 2010 to 2018. This is further classified into EOD versus LOD

Out of the 71 cases of invasive GBS disease, 59 isolates were sent for serotyping in the NPHL (Fig. 1). Serotype III, mainly in the form of serotype III ST17 or ST1340, was the most common serotype in both the EOD (n = 6, 37.5%) and LOD groups (n = 36, 65.5%; total n = 42, 71.2%). Serotype Ia was the second most common serotype identified (n = 9, 15.3%) followed by serotype V (n = 3, 5.1%). Serotypes Ia, Ib, II, III, and V accounted for 98.3% (n = 58) of the serotypes sent for testing while serotypes Ia, Ib and III (n = 53) were seen in 89.8% of the GBS isolates that we had identified. All the cases of meningitis (with or without concomitant bacteremia) were caused by serotype III (Table 2). All the GBS isolates were susceptible to penicillin.

**Table 2:** Serotype distribution leading to bacteraemia and meningitis in KKH, Singapore (2010–2018)

GBS serotypes^a^	Number (%) (n = 59)	Bacteremia without meningitis (%) (n = 45)	Meningitis with or without bacteremia (%) (n = 14)
Ia	9 (15.3)	9 (20.0)	0
Ib	2 (3.4)	2 (4.4)	0
II	2 (3.4)	2 (4.4)	0
III	42 (71.2)	28 (62.2)	14 (100)
V	3 (5.1)	3 (6.7)	0
VI	1 (1.7)	1 (2.2)	0

^a^12 missing serotype in 2014

### Incidence of EOD and LOD

The mean incidence of invasive GBS disease was 0.42 per 1000 live births (range: 0.08 to 0.59 per 1000 live births) (Table 3). The mean incidences of EOD and LOD were 0.13 per 1000 live births (range: 0 to 0.18 per 1000 live births) and 0.29 per 1000 live births (range: 0.08 to 0.51 per 1000 live births), respectively. The LOD incidence was at a mean of 2 times (range: 1.0 to 6.0) of EOD incidence per year. The number of LOD cases was consistently higher than EOD cases in each year of the study.

**Table 3:** Incidence of EOD and LOD in KK Women’s and Children’s Hospital, Singapore (2010–2018)

Year	Number of live births in KKH	Number of EOD	Number of LOD	Incidence of EOD	Incidence of LOD	Total incidence of EOD and LOD	LOD/EOD
				Per 1000 live births			
2010	11,271	2	4	0.18	0.35	0.53	2.0
2011	11,776	1	1	0.08	0.08	0.17	1.0
2012	11,794	1	6	0.08	0.51	0.59	6.0
2013	11,055	2	4	0.18	0.36	0.54	2.0
2014	11,782	2	3	0.17	0.25	0.42	1.5
2015	12,061	0	1	0.00	0.08	0.08	NA^a^
2016	11,894	2	3	0.17	0.25	0.42	1.5
2017	11,882	2	5	0.17^b^	0.42^b^	0.59^b^	2.5^b^
Mean	NA	NA	NA	0.13	0.29	0.42	2.0

*LOD* late onset disease, *EOD* early onset disease, *NA* not applicable

^a^No number as there were 0 EOD cases in 2015

^b^Incidence of EOD and LOD in 2017 is an underestimate as infants diagnosed with invasive GBS disease beyond October 2017 were not included in this study

### Risk factors of invasive GBS LOD

As compared to EOD, GBS LOD was associated with the Chinese ethnicity (69.1% in LOD vs 12.5% in EOD, OR 15.6, 95% CI 3.2–76.6, p < 0.001), negative/unknown maternal GBS status (92.7% in LOD vs 62.5% in EOD, OR 10.0, 95% CI 2.0–33.3, p = 0.007) and serotype III (65.5% in LOD vs 37.5% in EOD, OR 5.3, 95% CI 1.5–19.3, p = 0.007) (Table 1) in the univariate analysis. On multivariate analysis, both Chinese ethnicity and negative/unknown maternal GBS status were still found to be significantly associated with GBS LOD. Risk factors associated with LOD were: Chinese ethnicity (OR 27.1, 95% CI 3.0–243.1, p = 0.003) and negative/unknown maternal GBS status (OR 20.0, 95% CI 2.0–250.0, p = 0.012). Prematurity and intrapartum risk factors (peripartum maternal pyrexia and prolonged rupture of membrane) of EOD were not associated with LOD (Table 1).

## Discussion

This is the first study that showed the invasive GBS disease serotype distribution in Singapore. Serotype III was the most common serotype in both the EOD and LOD groups. Serotypes Ia, Ib, II, III, and V also accounted for 98.3% (n = 58) of the serotypes detected for invasive GBS diseases. We also showed that the mean incidence of invasive GBS disease was 0.42 per 1000 live births. The LOD incidence was at a mean of 2 times (range: 1.0 to 6.0) of EOD incidence per year despite the GBS preventive strategies. Known risk factors of EOD such as prematurity, peripartum maternal pyrexia and prolonged rupture of membrane were not associated with LOD. Chinese ethnicity and negative/unknown maternal GBS status were found to be associated with LOD as compared to EOD.

Worldwide, meta-analysis of serotype prevalence showed that GBS serotype III was the predominant serotype [10, 17, 18]. Around 50% of the EOD and 75% of the LOD cases were caused by serotype III. Five serotypes (Ia, Ib, II, III and V) were responsible for 97% of invasive GBS diseases around the world. Our findings mirrored this where serotype III was the most common isolate in our cohort and the serotypes Ia, Ib, II, III, and V accounted for 98.3% of the detected GBS isolates  in our cohort. Serotypes Ia, Ib and III were also frequently reported to cause invasive GBS disease around the world, and these three serotypes were seen in 89.8% of the GBS isolates that we had identified. A trivalent (serotype Ia, Ib and III) GBS conjugate vaccine or a hexavalent capsular polysaccharide conjugate vaccine GBS6 (serotypes Ia, Ib, II, III, IV and V) is likely to cover the majority of causative GBS serotypes in our population.

As compared to other Asian countries, our findings  were similar to studies from China [19, 20] and Taiwan [21] where serotype III was the most common serotype accounting for invasive GBS disease although in Taiwan, serotype VI was the leading cause of EOD from 2007 to 2010. It appears that serotypes leading to invasive GBS disease in infants can be similar although some variations can occur in different geographical locations, climate, and year of study. In our cohort, we also detected 1 EOD which was caused by serotype VI. This serotype does not lead to invasive disease commonly, although it had been reported to lead to significant proportions of maternal GBS colonization in Southeastern Asia countries previously [10]. We do not have the serotype data of maternal GBS colonization in our cohort to make this comparison.

The mean incidence of invasive GBS disease in our study was 0.42 per 1000 live births which was lower than the reported worldwide pooled incidence of 0.49 per 1000 live births in a systematic review [10]. In studies done in Asia, the overall incidence of GBS disease was 0.31 per 1000 live births and 1.1 per 1000 live births in China and Taiwan, respectively [19, 21]. Our mean EOD incidence of 0.13 per 1000 live births was also lower than the reported worldwide pooled EOD incidence of 0.41 per 1000 live births. There was a decrease in the incidence of EOD found in this study as compared to the previous finding of 0.265 per 1000 live births before the implementation of steps to reduce invasive GBS disease in our institution [9]. This shows that the burden of GBS EOD in our country had decreased over the years. However, the implementation of the current GBS preventive strategies did not have any impact on the LOD burden. Our mean LOD incidence remained high at 0.29 per 1000 live births. Similar to our findings, other studies also showed that the LOD rates were not affected by the widespread use of intrapartum antibiotic prophylaxis for high-risk pregnant women [1–3, 12, 22]. Twenty-five percent of our infants with LOD had GBS meningitis implying significant morbidity for this group of patients. In a systematic review, 32% of GBS meningitis survivors had neurodevelopmental impairment at 18 months of follow-up, including 18% with moderate to severe neurodevelopmental impairment [23]. New interventions such as GBS vaccine are required to tackle the GBS LOD burden in our country and worldwide.

The risk factors for EOD had been well described in many studies [2, 22, 24–29]. Prematurity, increased duration of rupture of membranes, maternal intrapartum fever, maternal vaginal GBS colonization and maternal GBS bacteriuria are proven risk factors of GBS EOD. However, the risk factors for LOD are less known. Maternal vaginal GBS colonization was reported to be a risk factor for LOD in a few studies, but these findings were inconsistent [5, 6]. On the other hand, prematurity was reported to be strongly associated with GBS LOD [3–6, 22]. In our cohort, only the Chinese ethnicity and negative/unknown antenatal maternal GBS status were associated with LOD after multivariate analysis. This association between LOD and Chinese ethnicity was not previously reported, although the African American ethnicity was associated with GBS LOD in some studies in the United States [5, 22, 30]. This association was thought to be due to socio-economic factors and access to medical care. The association between Chinese ethnicity and LOD in our study may reflect differences in cultural practices and should warrant further investigation. We do not have the socio-economic status of the infants in our population to evaluate the impact of socio-economic factors on GBS LOD.

We also found that LOD was associated with negative/unknown antenatal maternal GBS status, and this suggests that antenatal maternal GBS status is not helpful in estimating the risk of LOD. In settings where elective Caesarean section has been arranged, vaginal GBS screening swabs will not be performed by most Obstetricians since GBS antibiotics prophylaxis will not be recommended for the clinical setting. It is known that vaginal screening swabs may yield false negative result due to the transient GBS colonization in the vaginal tract [11]. Improper sampling can also lead to false negative results as rectovaginal swab for GBS gives the best yield as compared to vaginal swabs [31]. GBS screening is recommended to be done between 35 and 37 weeks of gestation in most guidelines and GBS colonization may still occur after the initial screening tests yield negative result. Moreover, there are other potential sources of GBS transmission from the mother to baby such as contaminated breastmilk with GBS that had been reported to be associated with GBS LOD [32]. It was suggested that the breastmilk might be contaminated through the transmission of GBS from the breastfed infants who had been initially colonized with GBS during delivery or after birth, leading to multiplication of GBS in the mammary ducts and persistent exposure of the infant and mother to GBS thereafter [33, 34]. The other proposed mechanism was the translocation of GBS from the gastrointestinal tract via the lymphatics to the mammary glands [32]. High bacterial load in cases of mastitis and prematurity with less developed immune function were two other factors associated with GBS LOD after the exposure to GBS contaminated breastmilk [5, 35]. At this point, it is not proven that GBS in breastmilk is indeed the causative factor for LOD and there is not enough evidence for breastfeeding to be discontinued if the breastmilk is found to be GBS positive. There is also no consensus regarding the routine testing of breastmilk for GBS. As our study is a retrospective analysis, we do not have the data to study these potential risk factors of GBS LOD.

Another reported risk factor for LOD was HIV exposure during the antenatal period [36, 37]. None of our infants were born to HIV positive mothers. Lastly, young maternal age and maternal consumption of capsules containing dehydrated placenta were reported to be associated with LOD in the United States in some studies [4, 21, 30, 38]. However, no mothers in our study reported consumption of these capsules and we do not have the maternal ages of our cohort to make this comparison.

Our study has some limitations. A small number of GBS isolates were obtained in the 8 years of the study and the isolates in the year 2014 were not available for serotyping. However, based on the fact that the distribution of serotypes in other years of the study had remained largely similar, it is likely that the serotype distribution in 2014 will reflect the trend found in other years. This is a single-centre study although our institution is the biggest tertiary neonatal and paediatric hospital. Based on our calculations, the reported EOD and LOD incidences are estimates for the entire country during the years of study. In our cohort, all of our infants survived the invasive GBS disease and those who recovered with no outstanding concerns were not given long-term follow up. Hence, we do not have long-term developmental follow up data on these infants who were infected with GBS. Based on an earlier study conducted in our institution from Jan 1998 to May 2013, 3 out of 20 (15%) of the GBS meningitis survivors developed moderate to severe neurodevelopmental impairment [39].

## Conclusion

Serotypes Ia, Ib, II, III, and V account for 98.3% (n = 58) of the invasive GBS diseases. The overall incidence of invasive GBS disease was 0.42 per 1000 live births, and the LOD incidence was at a mean of 2 times (range: 1.0 to 6.0) of EOD incidence per year. A GBS vaccine that covers the major causative serotypes found in our cohort can potentially reduce the overall GBS disease burden in the country.

## Data Availability

The datasets used and/or analyzed during the current study are available from the corresponding author on reasonable request.

## Abbreviations

GBS: Group B streptococcus
EOD: Early onset disease
LOD: Late onset disease
IQR: Interquartile range
n: Number
OR: Odds ratio
CI: Confidence interval
CDC: Centres for Disease Control and Prevention
USA: United States of America
RCOG: Royal College of Obstetricians and Gynaecologists
NICE: National Institute for Health and Care Excellence
KKH: KK Women’s and Children’s Hospital
CSF: Cerebrospinal fluid
NPHL: National Public Health Laboratory
PCR: Polymerase chain reaction
MLST: Multi-locus sequence typing
HIV: Human immunodeficiency virus
ECMO: Extracorporeal membrane oxygenation
